# Two Different Populations within the Healthy Elderly: Lack of Conflict Detection in Those at Risk of Cognitive Decline

**DOI:** 10.3389/fnhum.2017.00658

**Published:** 2018-01-11

**Authors:** Sergio M. Sánchez-Moguel, Graciela C. Alatorre-Cruz, Juan Silva-Pereyra, Sofía González-Salinas, Javier Sanchez-Lopez, Gloria A. Otero-Ojeda, Thalía Fernández

**Affiliations:** ^1^Departamento de Neurobiología Conductual y Cognitiva, Instituto de Neurobiología, Universidad Nacional Autónoma de México, Santiago de Querétaro, Mexico; ^2^Escuela Superior de Atotonilco de Tula, Universidad Autónoma del Estado de Hidalgo, Atotonilco de Tula, Mexico; ^3^Facultad de Estudios Superiores Iztacala, Universidad Nacional Autónoma de México, Tlalnepantla, Mexico; ^4^Escuela Superior de Tepeji del Río, Universidad Autónoma del Estado de Hidalgo, Tepeji del Río, Mexico; ^5^Department of Neuroscience, Biomedicine and Movement Sciences, University of Verona, Verona, Italy; ^6^Facultad de Medicina, Universidad Autónoma del Estado de México, Toluca, Mexico

**Keywords:** EEG, theta activity, inhibitory control, ERPs, counting-stroop task, aging, N500, N450

## Abstract

During healthy aging, inhibitory processing is affected at the sensorial, perceptual, and cognitive levels. The assessment of event-related potentials (ERPs) during the Stroop task has been used to study age-related decline in the efficiency of inhibitory processes. Studies using ERPs have found that the P300 amplitude increases and the N500 amplitude is attenuated in healthy elderly adults compared to those in young adults. On the other hand, it has been reported that theta excess in resting EEG with eyes closed is a good predictor of cognitive decline during aging 7 years later, while a normal EEG increases the probability of not developing cognitive decline. The behavioral and ERP responses during a Counting-Stroop task were compared between 22 healthy elderly subjects with normal EEG (Normal-EEG group) and 22 healthy elderly subjects with an excess of EEG theta activity (Theta-EEG group). Behaviorally, the Normal-EEG group showed a higher behavioral interference effect than the Theta-EEG group. ERP patterns were different between the groups, and two facts are highlighted: (a) the P300 amplitude was higher in the Theta-EEG group, with both groups showing a P300 effect in almost all electrodes, and (b) the Theta-EEG group did not show an N500 effect. These results suggest that the diminishment in inhibitory control observed in the Theta-EEG group may be compensated by different processes in earlier stages, which would allow them to perform the task with similar efficiency to that of participants with a normal EEG. This study is the first to show that healthy elderly subjects with an excess of theta EEG activity not only are at risk of developing cognitive decline but already have a cognitive impairment.

## Introduction

Due to the increase in life expectancy, the diseases associated with old age, such as dementia, are becoming more frequent (Harada et al., 2013); that is why there has been a growing interest in the study of this population. During aging, both physical and cognitive functions are naturally affected (Román Lapuente and Sánchez Navarro, 1998). The deterioration of cognitive functions in the elderly has been associated with structural and functional changes in the brain, which are considered typical of aging. EEG oscillations are strongly related to cognitive processes (Buzsáki, 2006; Lopes da Silva, 2011). Multiple studies have reported a relationship between global cognitive level and quantitative EEG analysis, showing a negative relationship between EEG slow activity at rest and scores in cognitive tests, in both healthy older adults (Roca-Stappung et al., 2012; Binder et al., 2017) and older adults with cognitive impairment (Prichep et al., 1994; Babiloni et al., 2006; van der Hiele et al., 2007; Kavcic et al., 2016).

According to Giaquinto and Nolfe (1986), a slower resting electroencephalogram (EEG) is characteristic of old age. This is manifested by a decrease in the frequency and amplitude of the occipital alpha rhythm, associated with a decrease in the posterior alpha power; the appearance of scattered theta waves, related to a diffuse increase in theta power; and the occasional presence of delta waves, mainly at temporal sites. Some authors (Chang et al., 2011), however, have proposed that changes in resting EEG that are attributed to aging could be the result of an ongoing subclinical pathological process and not the result of normal aging.

Several follow-up studies in the elderly (Soininen et al., 1991; Hartikainen et al., 1992; Huang et al., 2000; Jelic et al., 2000; Prichep et al., 2006) that included normal subjects, subjects with mild cognitive impairment (MCI), and subjects with dementia have been conducted. Some of them have noted that excessive activity in the theta frequency range of the resting EEG, with respect to an age-regressed normative database, is an excellent predictor of cognitive decline during aging (Prichep et al., 2006; van der Hiele et al., 2008). Prichep et al. (2006) recorded the EEG at rest with eyes closed in normal individuals who had a score of 2 on the Global Deterioration Scale (Reisberg et al., 1988); 10 years after, authors observed that more than 60% of the subjects had developed a cognitive decline and that their previous EEG already showed abnormal signs with respect to the norms, being the most relevant an excess of theta activity (4–7 Hz). The authors concluded that excess of theta activity in the EEG at rest with eyes closed of healthy older adults is the main electroencephalographic predictor of cognitive impairment in old age (Prichep et al., 2006). Using a logistic regression method, Prichep et al. (2006) obtained a coefficient of determination of 0.93 between the resting EEG features and the probability of future deterioration, with an overall prediction accuracy of 90%, which indicates a high sensitivity and specificity for the resting EEG values as predictors of the future state in normal subjects.

Taking into account this prediction, in a previous study in which we compared a group of healthy older adults with theta excess in their resting EEG to another group that had a normal EEG, we found differences in brain structure in their magnetic resonance images (MRI); we interpreted some of these differences as compensatory changes that could explain the similar cognitive performance of these two groups (Castro-Chavira et al., 2016). Then, the “healthy” elderly population may include, among others, two sub-groups that can be clearly differentiated: those with normal EEG and without risk and those with increased EEG theta activity. The latter seems to have developed a neural reorganization that significantly increases its probability to show cognitive impairment signs in the future despite not showing any clinical signs at the point of measurement. Although hypothesized, nobody has deeply explored whether the healthy elderly with theta excess are already experiencing some cognitive alteration beyond the natural process of healthy aging.

In general, healthy aging is related to a decline in specific cognitive functions, e.g., executive functions (EFs) (Buckner, 2004; Hedden and Gabrieli, 2004; Grieve et al., 2007; Schneider Bakos et al., 2008; Vaughan and Giovanello, 2010; Berry et al., 2016). In particular, cognitive processes, such as inhibitory control and attention, are affected (Thomas et al., 2010). Studies using event-related potentials (ERPs) have supported the inhibitory-deficit theory at the sensorial, perceptual, and cognitive levels during aging (Chambers and Griffiths, 1991; Alain and Woods, 1999; West and Alain, 2000). Differences between subjects with normal EEG and subjects with electroencephalographic risk of cognitive decline could be found in the brain response pattern using ERPs, especially in inhibitory control. These differences can indicate how cognitive impairment begins to be expressed in structural and functional changes in the central nervous system, although differences are not yet clinically observable. When a subject performs a task, behavioral results, which are typically measured as percentages of correct responses or reactions times, are the consequence of multiple processes organized sequentially or in parallel. ERPs provide the best temporal resolution of cognitive processing; therefore, they constitute an excellent tool to detect deficits in any of the sub-processes needed to solve the task.

To study inhibitory control, Stroop tasks (Stroop, 1935) have been used. Stroop tasks are characterized by measuring selective attention and the inhibition of automatic responses (Bush et al., 1998, 2006). Studies using Color- or Counting-Stroop tasks have been carried out to explore the dynamics of inhibitory cognitive processing (West and Alain, 1999; West et al., 2005, 2012). Specifically, in the Counting-Stroop task, subjects are asked to answer how many words are presented in a slide, regardless of the meaning of the word itself. Subjects increase their response times and tend to make more mistakes when the meaning of the word does not match with the number of times that the word appears; this phenomenon is known as the Stroop or interference effect (MacLeod, 1991). This effect appears because two processes are in competition—an automatic process, which is generated by reading, and the counting process—this effect is increased in older adults as a result of a decline in inhibitory process efficiency (West and Alain, 2000). A negative wave located on frontal-central regions (referred as N450 in young adults or N500 in older adults) is observed during the Counting-Stroop task, and it has been related to a response of interference processing (West and Alain, 1999); the N500 effect reflects an active inhibitory process and is related to suppression or attenuation of semantic word information upon the response selection processes (West and Alain, 1999, 2000). Aging seems to be associated with a greater Stroop effect, i.e., an increase in the difference between the reaction times from one condition to the other, in favor of the incongruent (Spieler et al., 1996; West and Alain, 2000; Zysset et al., 2007), a lower amplitude of the N500 component, and a lower N500 effect, i.e., a reduction in the difference between conditions in the time-window corresponding to the N500 component (West and Alain, 2000).

The aim of this study was to explore whether healthy older adults with an electroencephalographic risk of cognitive impairment (i.e., resting EEG theta-activity excess), show lower inhibitory control than healthy older adults of the same age whose EEG was normal. This question was assessed by ERPs recorded during a Counting-Stroop task. Considering that aging is associated with cognitive deterioration, we expected to find more pronounced, age-related, cognitive deficits in the group at risk of cognitive decline, i.e., lower N500 amplitude, a lower N500 effect, and a higher Stroop effect.

## Materials and methods

### Participants

Forty-four right-handed healthy older adults aged over 60 years (26 females) with a score of 1 or 2 in the Global Deterioration Scale (GDS; Reisberg et al., 1988, 2008), scores between 90 and 109 for the Wechsler Intelligence Scale for adults (WAIS-III; Wechsler, 2003), more than 9 years of schooling, and more than 70% in the Q-LES-Q questionnaire (Endicott et al., 1993) participated in this study (Table 1). All participants were within normal limits in the brief version of a neuropsychological battery normalized in Mexican population (NEUROPSI; Ostrosky-Solís et al., 1999) and they showed no evidence of a neurological or psychiatric disorder. Psychiatric illnesses were discarded by Mini-Mental State Examination (MMSE; > 27), GDS, Alcohol Use Disorders Identification Test (AUDIT; Babor et al., 2001; < 5), Beck Depression Inventory (Beck et al., 1961; < 4), the Geriatric Depression Scale (Yesavage et al., 1983; < 5), and a psychiatric interview. Neurological disorders were discarded by a clinical interview and a neurological physical examination. Both evaluations were performed by a geronto-psychiatrist.

**Table 1:** Characteristics of the samples.

	**Group**	**Mean ± sd**	***t* (42)**	***p*-value**
Age (years)	Normal	65.54 ± 5.21	−1.22	0.22
	Theta	67.59 ± 5.81		
IQ (WAIS)	Normal	103.78 ± 7.95	0.84	0.40
	Theta	101.74 ± 8.50		
Scholar Education (years)	Normal	15.81 ± 5.13	0.36	0.71
	Theta	15.31 ± 3.84		
Q-LES-Q score	Normal	79.26 ± 8.04	0.35	0.72
	Theta	78.21 ± 10.98		
AUDIT score	Normal	2.00 ± 1.95	−0.16	0.87
	Theta	2.09 ± 1.77		

Furthermore, subjects did not present signs of diabetes, anemia, hypercholesterolemia, or thyroid disease in clinical blood analysis, nor did they have uncontrolled hypertensive disease. The participants and two witnesses signed informed consent.

This project was approved by the Bioethics Committee of the Neurobiology Institute of the National Autonomous University of Mexico (UNAM).

These 44 subjects were classified into two groups according to the characteristics of their EEG: the Theta-EEG group, in which subjects presented an excess of theta activity for their age in at least one electrode, and the Normal-EEG group, in which subjects presented normal EEGs, from both the qualitative and quantitative point of view.

### EEG

To identify which subjects belonged to the Normal-EEG and the Theta-EEG groups, an EEG was recorded from each participant. The digital EEG was recorded at rest with eyes closed using a Medicid™ IV system (*Neuronic Mexicana, S.A*.; Mexico) and Track Walker TM v5.0 data system for 15 min from 19 tin electrodes (10–20 International System, ElectroCap™, International Inc.; Eaton, Ohio) referenced to linked ear lobes. The EEG was digitized using the MEDICID IV System (Neuronic A.C.) with a sampling rate of 200 Hz using a band pass filter of 0.5–50 Hz, and the impedance was kept below 5 kΩ. To assess the EEG, visual inspection and quantitative EEG (QEEG) analyses carried out by a clinical neurophysiologist were considered.

Twenty-four artifact-free segments of 2.56 s each were selected, and the QEEG analysis was performed offline using the Fast Fourier Transform to obtain the power spectrum every 0.39 Hz; also the geometric power correction (Hernández et al., 1994) was applied and absolute (AP) and relative power (RP) in each of the four classic frequency bands were obtained: delta (1.5–3.5 Hz), theta (3.6–7.5 Hz), alpha (7.6–12.5 Hz), and beta (12.6–19 Hz); these frequency ranges were the same as those used for the normative database (Valdés et al., 1990) provided by MEDICID IV. *Z*-values were obtained for AP and RP, comparing subject's values with values of the normative database [Z = (x–μ)/σ, where μ and σ are the mean value and the standard deviation of the normative sample of the same age as the subject, respectively]; *Z*-values >1.96 were considered abnormal (*p* < 0.05).

### Groups

The Normal-EEG group, which included 22 participants with normal EEGs (65.54 ± 5.21 y.o., 13 females), and the Theta-EEG group, with 22 participants with abnormally high *Z*-values of theta AP (Z > 1.96) on at least one electrode (67.59 ± 5.81 y.o., 13 females), did not differ in age, Intelligence Quotient (IQ), number of scholar years, percentage in the Q-LES-Q or the AUDIT scale for substance abuse (Babor et al., 2001). The groups differed only in terms of their EEG. Table 1 shows *t*- and *p*-values obtained by Student's *t*-test for independent samples used for the comparison between groups.

### Counting-stroop task

Series of one, two, three, or four words that denote numbers (“one,” “two,” “three,” “four”) were presented in the center of a 17-inch computer screen. Time presentation of the stimuli was 500 ms, and the inter-stimulus interval was 1,500 ms. An incongruent condition consisted in a trial where the number of presented words did not correspond with the meaning of the word; the congruent condition consisted in a trial in which the number of presented words and the meaning of the word that was presented matched. A total of 120 incongruent and 120 congruent stimuli were randomly presented.

Subjects were asked to indicate the number of times that the word appeared in each trial, using a response pad that they held in their hands. One-half of the participants used their left thumbs to answer “one” or “two” and their right thumbs to indicate “three” or “four”; the other half of the participants used their opposite hand to counterbalance the motor responses. The participants were asked to answer as quickly and accurately as possible. We ensured that the participants understood the instructions by presenting a brief practice task before the experimental session.

### ERP acquisition and analysis

The EEGs were recorded with 32 Ag/AgCl electrodes mounted on an elastic cap (Electrocap), using NeuroScan SynAmps amplifiers (Compumedics NeuroScan) and the Scan 4.5 software (Compumedics NeuroScan). Electrodes were referenced to the right earlobe (A2), and the electrical signal was collected from the left earlobe (A1) as an independent channel. Recordings were re-referenced off-line in two ways: (a) to the averaged earlobes, as usually was performed in previous studies, and (b) to the average reference (see Supplementary Material). The EEG was digitized with a sampling rate of 500 Hz using a band pass filter of 0.01 to 100 Hz. Impedances were kept below 5 kΩ. An electrooculogram was recorded using a supraorbital electrode and an electrode placed on the outer cantus of the left eye.

ERPs were obtained for each subject and experimental condition (i.e., congruent and incongruent). Epochs of 1,500 ms were obtained for each trial that consisted of 200-ms pre-stimulus and 1,300-ms post-stimulus intervals. An eye movement correction algorithm (Gratton et al., 1983) was applied to remove blinks and vertical ocular-movement artifacts. Low pass filtering for 50 Hz and a 6-dB slope was performed offline. A baseline correction was performed using the 200-ms pre-stimulus time window, and a linear detrend correction was performed on the whole epoch. Epochs with voltage changes exceeding ±80 μV were automatically rejected from the final average. The epochs were visually inspected, and those with artifacts were also rejected. Averaged waveforms for each subject and each type of stimulus included only those trials that corresponded to correct responses.

### Statistical analysis

#### ERP behavioral analysis

Three mixed, 2-way ANOVAs were separately performed for the mean reaction time, reaction time variability, and percentage of correct answers. The between-subject factor was group (Normal-EEG and Theta-EEG), and the within-subject factor was condition (congruent and incongruent). The percentages of correct responses were transformed using the function {ARCSINE [Square Root (percentage/100)]} to ensure normal distribution of the data (McDonald, 2009). Tukey's honest significant difference (HSD) *post-hoc* tests were performed for multiple comparisons.

#### ERP amplitude analysis

**Figure 1:**
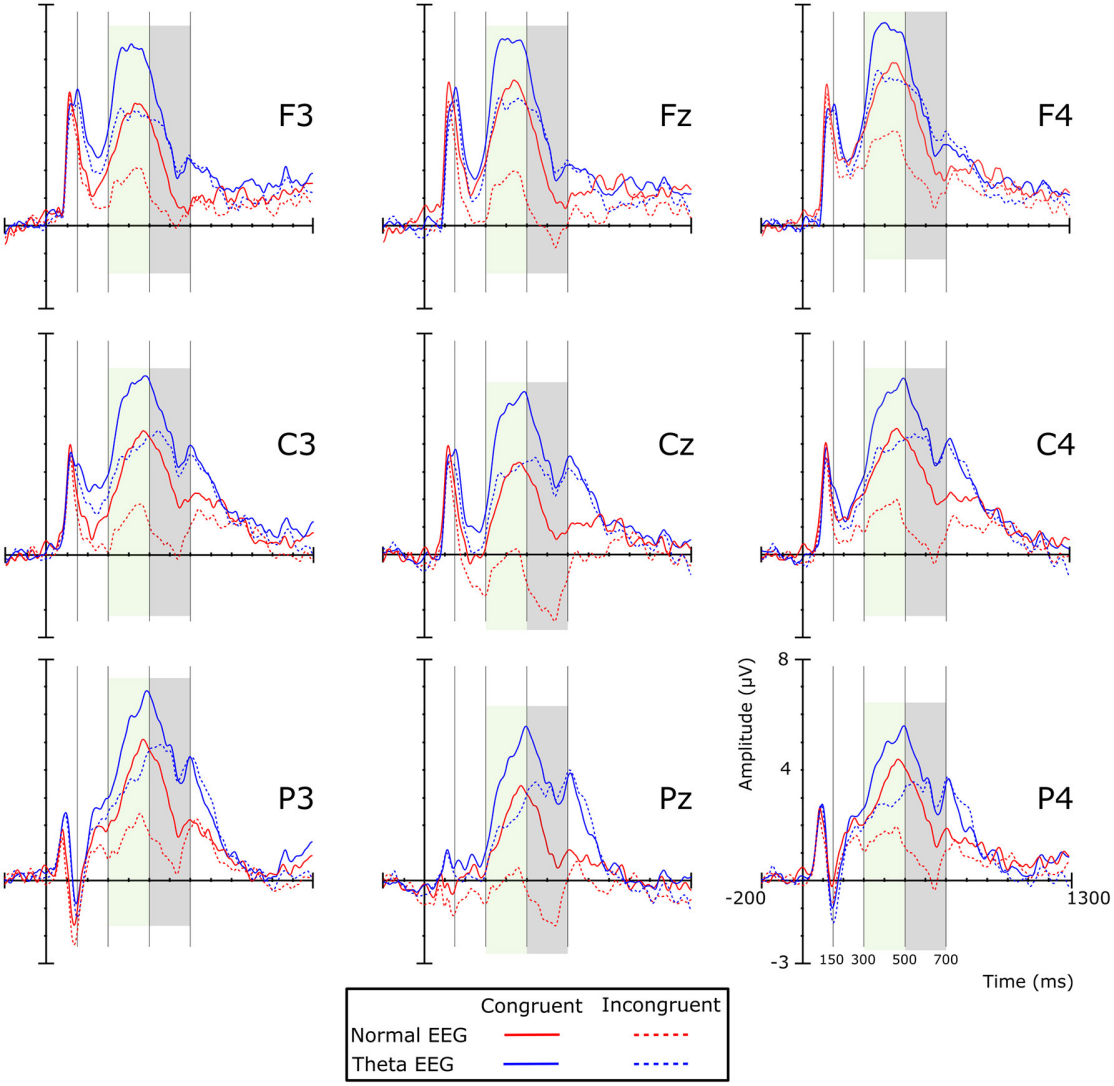
Grand average ERPs per experimental condition by group. Colored shadow boxes indicate significant differences between the conditions in the same group. Positive amplitude is plotted upward. Red lines represent ERPs of the Normal-EEG group, and blue lines represent the Theta-EEG group. Solid and dotted lines represent congruent and incongruent conditions, respectively.

**Figure 2:**
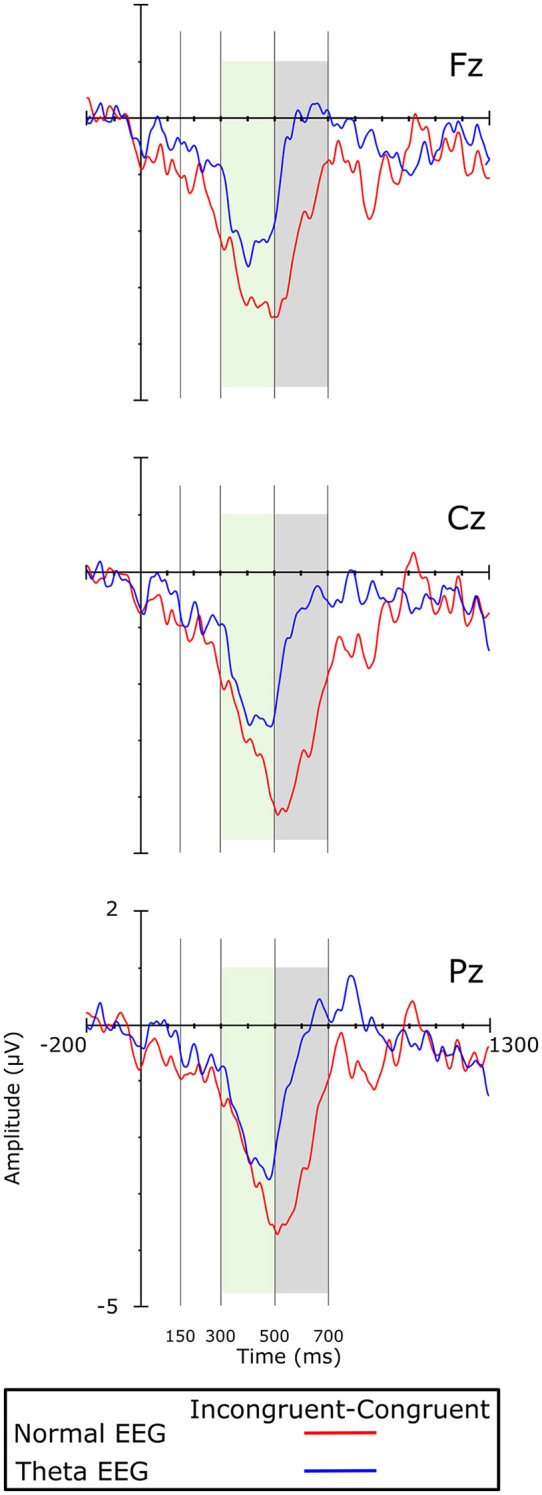
Difference waves (i.e., incongruent minus congruent condition) on the midline electrodes. Colored boxes indicate significant differences between the groups.

Visual inspection of the grand averages of the ERPs (Figure 1) showed a peak at ~250 ms on the frontal regions for both groups. A brainwave associated with the congruent condition was smaller (i.e., less negative) than those associated with the incongruent condition; such negative deflection will be referred to as the N200 component. This wave was followed by a positive deflection that started at ~300 ms post-stimulus and lasted ~200 ms, showing larger amplitudes for the congruent than for the incongruent condition. According to their appearance in the grand average waveforms, this was considered a P300 component. The next important wave observed in the grand average of the ERPs was a negativity peaking between 500 and 700 ms, which was mainly observed in the Normal-EEG group; it was designated as the N500 component. The mean amplitude was statistically analyzed and computed as the average of the voltages within the 150–300, 300–500, 500–700, and 850–1,150-ms intervals, according to the literature (West and Alain, 2000; West et al., 2012). The difference wave was calculated by subtracting the congruent condition from the incongruent condition and is shown in Figure 2.

Four mixed, 4-way ANOVAs were independently performed on the mean amplitude data, one per each ERP component. Group [Normal-EEG and Theta-EEG] was included as the between-subjects factor. The within-subjects factors were: (a) Condition [Congruent and Incongruent], (b) Coronal [Frontal (F7, F3, Fz, F4, and F8), Fronto-central (FT7, FC3, FCz, FC4, and FT8), Central (T3, C3, Cz, C4, and T4), Central-parietal (TP7, CP3, CPz, CP4, and TP8), and Parietal (T5, P3, Pz, P4 and T6)]; and (c) Sagittal [Left (F7, FT7, T3, TP7, and T5), Medial-left (F3, FC3, C3, CP3, and P3), Medial (Fz, FCz, Cz, CPz, and Pz), Medial-right (F4, FC4, C4, CP4, and P4), and Right (F8, FT8, T4, TP8, and T6)]. The Huynh-Feldt correction was applied to the degrees of freedom of those analyses with more than one degree of freedom in the numerator. Degrees of freedom are reported uncorrected, but the epsilon value was included. Only differences that involved the main effect of Group and any interaction of Group by Condition are reported. Tukey's HSD *post-hoc* tests were performed for multiple comparisons.

The same analysis was also performed when the average reference was used, and it is described in the Supplementary Material.

## Results

### ERP behavioral results

The mean reaction times (RTs), RTs variability, and percentage of correct responses for the congruent and incongruent conditions of the two groups are shown in Table 2.

**Table 2:** Mean and standard deviation (SD) of the reaction times and percentage of correct responses.

**Group**	**N**	**Stimulus**	**Mean RTs (Mean ± sd)**	**RTs Variability (Mean ± sd)**	**% Correct responses (Mean ± sd)**
Normal EEG	22	Incongruent	721.80 ± 53.83	11462.90 ± 2410.50	79.05 ± 12.05
		Congruent	652.88 ± 52.61	10569.93 ± 2853.51	85.94 ± 8.69
Theta EEG	22	Incongruent	702.12 ± 72.60	11501.90 ± 2941.84	77.72 ± 13.07
		Congruent	649.62 ± 65.25	10901.97 ± 4454.62	86.17 ± 9.37

*RTs: Reaction Times*.

Although there was no significant main effect of Group (*F* < 1) when the mean RTs was analyzed, there was a significant Group × Condition interaction [*F*_(1, 42)_ = 5.408, *p* = 0.025, η^2^*p* = 0.114]. *Post-hoc* comparison tests showed a greater Stroop effect (i.e., longer RTs to the incongruent than to the congruent condition) for the Normal-EEG group (Mean Difference (MD) = 68.92, *p* < 0.001) than for the Theta-EEG group (*MD* = 52.5, *p* < 0.001). The two-way ANOVA for the transformed percentage of correct responses showed no significant differences between the groups (main effect of Group (*F* < 1) and no significant Group × Condition interaction (*F* < 1). No significant main effect of Group (*F* < 1) or Condition [*F*_(1, 42)_ = 2.6, *p* = 0.11, η^2^*p* = 0.06] was observed, and no significant effect of Group × Condition interaction was found (*F* < 1) when the RTs variability was analyzed.

### ERP results

When common average re-referencing was used, the most critical results remained unchanged: no differences were found in N200, P300 effect was robust in both groups, and in the Normal-EEG group a great N500 effect was observed while in the Theta-EEG group this effect was absent. Therefore, we think, in general terms, that our results are almost not affected by the volume conduction. Taking into account that the global average from a limited number of channels might not be a good estimate of volume conduction, we only present the results when A1A2 reference was used.

#### 150–300 ms

No significant main effect of Group was observed for this time window [*F*_(1, 42)_ = 2.197, *p* = 0.146, η^2^*p* = 0.05], and the Group × Condition interaction was not significant (*F* < 1) either. When the topography factor was included, no significant differences were found (Group × Condition × Coronal interaction: *F* < 1; Group × Condition × Sagittal interaction: *F* < 1). The Group × Condition × Coronal × Sagittal interaction was also not significant [*F*_(16, 672)_ = 2.156, *p* = 0.074, ε = 0.257, η^2^*p* = 0.049].

#### 300–500 ms

**Figure 3:**
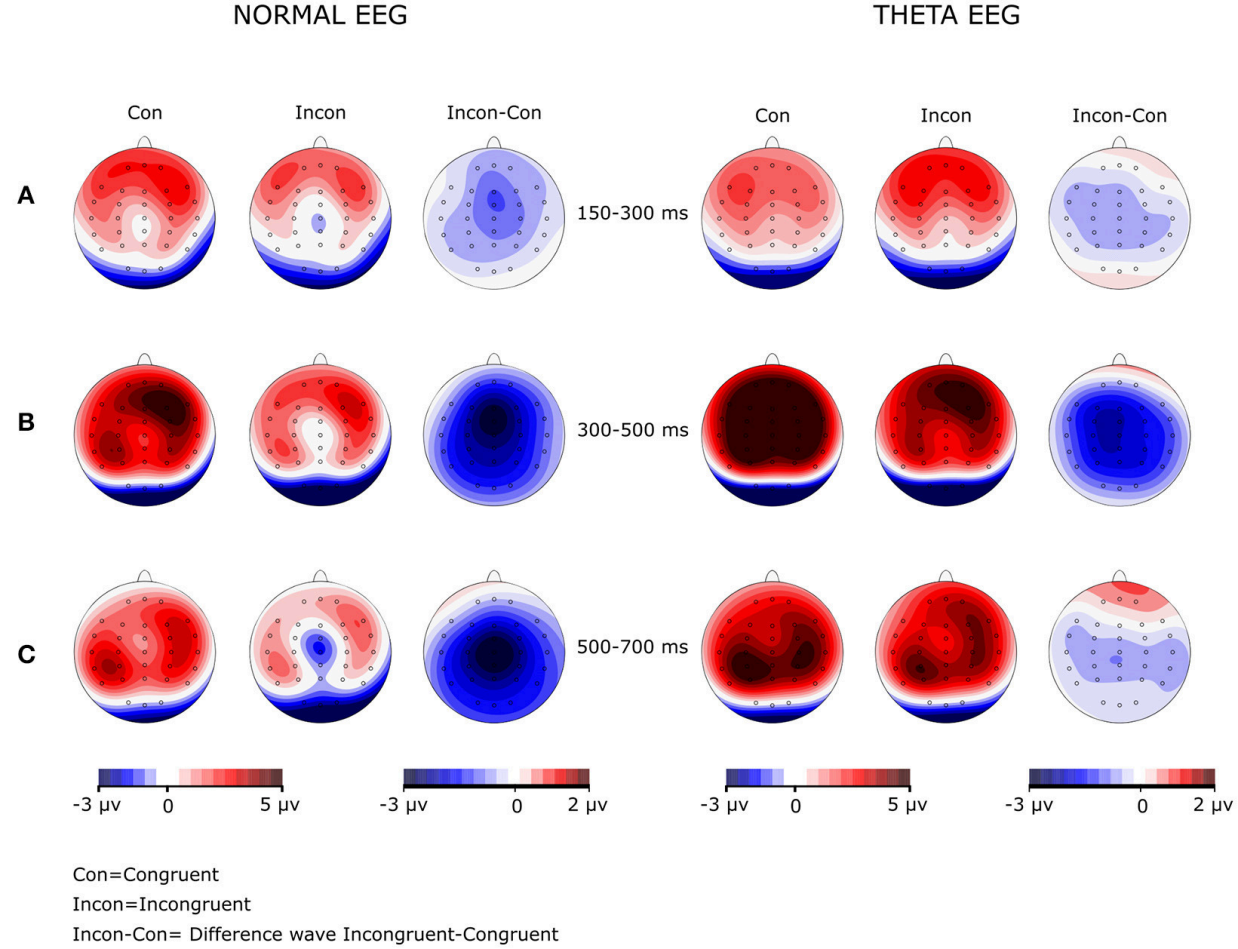
Amplitude maps and wave difference map in both groups at the three windows analyzed. The Normal-EEG group is on the left, and the Theta-EEG group is on the right. Note in the difference maps (Incon-Con) that **(A)** no N200 effect was observed for any group, **(B)** the P300 effect was generalized for both groups, and **(C)** there was a generalized N500 effect in the Normal-EEG group and a lack of an N500 effect in the Theta-EEG group.

The analysis of the amplitude in this time window showed a significant main effect of Group [*F*_(1, 42)_ = 7.163, *p* = 0.011, η^2^*p* = 0.584], with the amplitude for the Theta-EEG group (Mean = 4.287 μV) being greater than the one observed for the Normal-EEG group (Mean = 2.07 μV). There were no significant differences in the Group × Condition interaction (*F* < 1), in the Group × Condition × Coronal interaction (*F* < 1) or in the Group × Condition × Sagittal interaction (*F* < 1). Nevertheless, there was a significant Group × Condition × Coronal × Sagittal interaction [*F*_(16, 672)_ = 3.054, *p* = 0.014, ε = 0.28, η^2^*p* = 0.068]. *Post-hoc* comparisons showed that for most of the electrodes, the effect was significant in both groups. In the Normal-EEG group, significant differences between the conditions were observed in all electrodes (*p* < 0.05), except in F7 (*p* = 0.099), T4 (*p* = 0.107), and TP8 (*p* = 0.086), while in the Theta-EEG group, significant differences were observed in all electrodes (*p* < 0.05), except in F8 (*p* = 0.317) and TP7 (*p* = 0.085) (see Figure 1). The topographic maps in Figure 3 show that the amplitude in the congruent condition was greater than the amplitude in the incongruent condition for both groups in most of the locations.

#### 500–700 ms

In this time window, the amplitude of the negative wave showed a significant main effect of Group [*F*_(1, 42)_ = 8.038, *p* = 0.007, η^2^*p* = 0.161], and the amplitude was significantly greater in the Normal-EEG group than in the Theta-EEG group for both conditions. There was a significant Group × Condition interaction [*F*_(1, 42)_ = 4.974, *p* = 0.031, ε = 1.0, η^2^*p* = 0.106]: the Normal-EEG group displayed a significant effect [Mean Difference (MD) = Incongruent-Congruent = −1.772 μV, *p* < 0.001] that was not observed in the Theta-EEG group (*MD* = −0.277 μV, *p* = 0.563).

Group and Condition did not interact significantly with the Coronal distribution factor [*F*_(4, 168)_ = 1.27, *p* = 0.285, ε = 0.457, η^2^*p* = 0.0129], but they did interact significantly with the Sagittal factor [*F*_(4, 168)_ = 4.859, *p* = 0.01, ε = 0.492, η^2^*p* = 0.104]. The Normal-EEG group showed a significant interference effect in the Left (*MD* = −1.094 μV, *p* = 0.017), Left-medial (*MD* = −1.984 μV, *p* = 0.001), Medial (*MD* = −2.552 μV, *p* < 0.001), Right-medial (*MD* = −2.067 μV, *p* < 0.001), and Right (*MD* = −1.162 μV, *p* = 0.012) sagittal regions. In contrast, the Theta-EEG group did not show a significant interference effect in any sagittal area. A similar pattern was observed for the significant Group × Condition × Coronal × Sagittal interaction [*F*_(16, 672)_ = 3.277, *p* = 0.008, ε = 0.301, η^2^*p* = 0.072].

Table 3 shows that the *post-hoc* comparisons between the stimuli for the N500 wave across electrodes over the scalp (Figure 1) were significant for most of the locations in the Normal-EEG group, while for the Theta-EEG group, there were no significant differences at any location. The topographic maps (Figure 3) also show this N500 effect (i.e., the amplitude of the incongruent condition is higher than the amplitude of the congruent condition) across almost all electrodes over the scalp in the Normal-EEG group, while the Theta-EEG group does not display a significant N500 effect for any electrode.

**Table 3:** N500 (500–700 ms) *post-hoc* comparisons of Group × Condition × Coronal × Sagittal interaction.

**Coronal**	**Sagittal**	**Electrode**	**Normal-EEG**	**Theta-EEG**
			**(MD)**	**(MD)**
Frontal	Left	F7	−0.48	−0.42
	Left-medial	F3	−1.26^*^	0.20
	Medial	Fz	−1.86^**^	0.03
	Right medial	F4	−1.62^**^	0.28
	Right	F8	−0.99	0.57
Fronto–central	Left	FT7	−0.94	−0.35
	Left-medial	FC3	−1.85^**^	−0.26
	Medial	FCz	−2.59^***^	−0.30
	Right-medial	FC4	−1.89^**^	−0.10
	Right	FT8	−1.03^*^	−0.65
Central	Left	T3	−1.08^*^	−0.32
	Left-medial	C3	−2.34^***^	−0.45
	Medial	Cz	−3.03^***^	−0.50
	Right-medial	C4	−2.97^***^	−0.29
	Right	T4	−1.15^*^	−0.64
Centro-parietal	Left	TP7	−1.40^***^	−0.09
	Left-medial	CP3	−2.40^***^	−0.42
	Medial	CPz	−2.74^***^	−0.54
	Right-medial	CP4	−2.35^***^	−0.39
	Right	TP8	−1.23^**^	−0.41
Parietal	Left	T5	−1.57^**^	−0.25
	Left-medial	P3	−2.07^***^	−0.21
	Medial	Pz	−2.54^***^	−0.28
	Right-medial	P4	−2.01^***^	−0.21
	Right	T6	−1.43^**^	−0.44

* *p < 0.05*,

** *p < 0.01*,

*** *p < 0.001*.

*MD, Mean difference*.

## Discussion

The aim of this study was to explore the inhibitory control process, using ERPs, of healthy older adults with an electroencephalographic risk of cognitive impairment (i.e., an excess of theta activity in their EEG), considering a control group of older adults with normal EEG, during the performance of a Counting-Stroop task. We expected to find that adults with an excess of theta activity would show a greater detriment of inhibitory control with respect to healthy older adults of the same age with a normal EEG, displaying a greater Stroop effect (in behavioral measures) and smaller N500 amplitude and N500 effect (i.e., larger differences between ERP amplitude of the incongruent vs. congruent condition, with a greater amplitude for the incongruent condition).

### Behavioral evidence

Considering that all our subjects were physically healthy without signs of cognitive impairment and that the only difference between the groups was their EEG activity (normal or theta excess), one would expect that (a) both groups would show Stroop effects, and (b) the cognitive processing would be equal in the two groups or, if different, that there were advantages for the Normal-EEG group; that is, an increased Stroop effect would occur in the Theta-EEG group.

Our results confirm the first assumption: both groups displayed a Stroop effect; however, in contrast to the hypothesis that individuals at risk for cognitive decline would present higher Stroop effects (greater RTs in the incongruent than in the congruent trials as an effect of the interference), our results showed a significantly greater Stroop effect in the Normal-EEG group than in the Theta-EEG group. This result is not in accordance with the results obtained by other authors (Spieler et al., 1996; Zurrón et al., 2014) who reported increases in the Stroop effect as a consequence of aging and have explained this occurrence as a decline in the ability of older adults to inhibit competing word information during the incongruent trials.

Surprisingly, the Theta-EEG group had smaller mean RTs than the Normal-EEG group, which was more evident in the incongruent condition, although a significant level was not reached. The normal group appeared to perform worse on the task; however, this performance will be discussed further in the subsequent sections.

It is also important to consider that no differences between the groups were found in terms of the number of correct answers. This fact and the high percentages of correct answers suggest that both groups performed well on the task.

Another fact that should be considered is the greater standard deviation in the RTs observed in the Theta-EEG group than in the Normal-EEG group, which indicates less response-consistency between the subjects of the Theta-EEG group, which could be a sign of failures in the activation of automatic mechanisms underlying the conflict in several subjects at risk for cognitive decline. However, no differences in terms of RTs variability were observed between groups. Previous studies regarding intra-individual cognitive variability have pointed out that, as age increases so does the RTs variability (Bielak et al., 2010), and this makes possible to distinguish both between young and old adults (Hultsch et al., 2002) and between healthy and MCI elderly subjects (Dixon et al., 2007). In our study, however, the RTs variability was not able to distinguish between healthy seniors without risk (Normal-EEG) and with risk (Theta-EEG) of cognitive decline. Although the RTs variability has been associated with increased risk to dementia up to 6 years later (Holtzer, 2008; Anderson et al., 2016), this prediction does not seem to coincide with the prediction based on the EEG.

### ERP evidence

An interference effect was manifested on the ERPs as an ERP effect (i.e., the magnitude resulting from subtracting the amplitude of the incongruent stimulus minus the amplitude of the congruent stimulus) in several time windows that are linked to different processing stages in the Stroop effect, which is specifically discussed below for each time window.

#### 150–300 ms

The amplitude differences between conditions that have been reported in this window have been related to a cerebral response to a non-perceptual conflict that has been described in flanker tasks (van Veen and Carter, 2002); however, in Stroop tasks, there is no clear evidence of this effect (West and Alain, 2000). In our Counting-Stroop task, we did not expect to find differences between the conditions, which was confirmed by our results and suggests that both groups may be reflecting a similar attentional state (Patel and Azzam, 2005).

#### 300–500 ms

Although some authors exclude differences in the P300 component as a brain response related to the conflict (Larson et al., 2014), it is difficult to separate it in our study due to the robust response observed in both groups in the Counting-Stroop task. Amplitude in this time window is sensitive to the categorization of words as congruent or incongruent depending on their relationship with the number the words displayed (Zurrón et al., 2009). Our results showed that the amplitude of deflections of the Theta-EEG group was greater than those displayed by the Normal-EEG group for both conditions, which could be interpreted as a greater use of available resources dedicated to the categorization process in the Theta-EEG group (Ramos-Goicoa et al., 2016); in contrast, previous studies have shown that in older adults the P300 amplitude is reduced compared to younger participants (Zurrón et al., 2014).

In terms of the topographical distribution of the P300 component (Figure 3), Zurrón et al. (2009, 2014) observed that the P300 amplitude was maximal at the frontal locations in older participants and that it was maximal at the parietal locations in young participants. Our results are in agreement with this fact because both elderly populations of our study had maximum P300 amplitude at the frontal locations. However, Zurrón et al. (2014) did not find the P300 effect in their group of elders, and we did in both groups of old people, although the pattern of effects was different for each group. Regarding our hypothesis, we should have observed a more “frontalized” distribution in subjects with an electroencephalographic risk for cognitive decline, but our participants with normal EEG showed a pattern with more “frontalized” distribution. This finding could be a consequence of some compensatory phenomenon. Cabeza et al. (2002), using PET, studied the differences between two groups of older adults, one of good responders and another of poor responders in two memory tasks; they found that the activation pattern of the latter was more similar to that of young people. This fact has been interpreted as a necessity of developing compensatory mechanisms during aging in order to be able to have a better performance. Since the P300 is “frontalized” in the elderly, its amplitude is reduced in parietal regions (Ila and Polich, 1999), which is consistent with the posterior–anterior shift in aging (PASA) hypothesis (Davis et al., 2008). These findings are relevant to this study, given that some studies have suggested that cognitive processes supported by the frontal cortex are particularly vulnerable to the effects of aging (Samson and Barnes, 2013; Yuan and Raz, 2014).

#### 500–700 ms

In contrast to what was observed in the previously discussed time window, the N500 deflections had more amplitude in the Normal-EEG group than in the Theta-EEG group for the two conditions. The effect observed between 500 and 700 ms corresponds with the beginning of mechanisms involved in inhibitory processes to solve the conflict when this exists (West and Alain, 2000; Chen et al., 2011). Therefore, the Normal-EEG group appeared to allocate more resources to inhibit the interference that the semantic process produced over the counting.

Another important finding observed in the 500–700 ms time window was the presence of an N500 effect in almost all regions in the Normal-EEG group, as opposed to the lack of an N500 effect in the Theta-EEG group. This N500 effect has been associated with the inhibition of the competence between semantic and counting information (Rebai et al., 1997; West and Alain, 1999, 2000; Liotti et al., 2000; West, 2004; West et al., 2005; Mager et al., 2007; Coderre et al., 2011; Tillman and Wiens, 2011). On the other hand, the N500 effect that we observed had a wider distribution than that observed in many previous studies, where the N500 effect was located in the centro-parietal regions (Rebai et al., 1997; West and Alain, 1999, 2000; West, 2003, 2004; West et al., 2005; Mager et al., 2007). This different topography of the N500 effect, which was a generalized in older people with a normal EEG, likely reflects the involvement of other brain areas in the inhibition with the purpose of recruiting more resources in order to respond correctly.

#### Overview

In the early stages of processing in the Counting-Stroop task, there did not appear to be significant differences between the groups, as both groups seemed to recruit the same amount of resources for attention processing. Differences started to occur with more complex and demanding processes, such as the categorization process. Older adults with a risk of cognitive decline appeared to require more resources to categorize words as congruent or incongruent, and they did not perform the categorization process as well as older adults without a risk.

This finding was supported by the most dramatic and unexpected result of the present study: the lack of an N500 effect in the Theta-EEG group, while the normal EEG group exhibited a robust N500 effect distributed throughout the entire scalp. The N500 in the Stroop tasks has been related to response interference processing (West and Alain, 2000; Chen et al., 2011). Therefore, our results suggest that subjects in the Normal-EEG group detected the conflict between reading and counting and inhibited the reading to give correct answers, which is expected in a Stroop task; however, in the Theta-EEG group, the conflict between reading and counting probably was not adequately detected. Assuming that this is what is expected in a Stroop task, these subjects should not have answered correctly; however, they did. One explanation for this surprising finding not previously described in the literature, in our knowledge, is that subjects in the Theta-EEG group suppressed some cognitive operations in a strategy of the economy of stages (Park et al., 1996) or would show deterioration of the pattern of ocular movements during reading as a consequence of the orbitofrontal atrophy observed during aging (Rayner et al., 2006) or they could fail in the direct route of reading (Federmeier et al., 2010; Wlotko et al., 2012). Any of these three factors, or a combination of them, could lead to abnormal processing of reading, and it is reasonable to assume that if these factors are related to aging, they are more pronounced in the group of older adults who are at risk of cognitive impairment. The lack of an N500 effect in the Theta-EEG group, since the subjects responded correctly, leads us to think that they probably did not have to inhibit the meaning of the word at all, and this is possible if they failed in a previous process, such as semantic access; in this way, it is even possible to explain why their responses were faster. To ensure that there is a deficit in semantic processing in these individuals, and to make a more reliable hypothesis about why they did not exhibit an N500 effect, it would be necessary to carry out a study of ERPs during the performance, for example, of a task involving semantic priming.

Another plausible mechanism that could explain the lack of conflict between reading and counting is that in the Theta-EEG group, the reduction in processing speed, a characteristic of aging (Salthouse, 1996, 2000; Baltes and Lindenberger, 1997; Verhaeghen et al., 2003), has been more severe, and as a consequence, the subjects did not have time to read the words in the 500 ms allowed to do so. This lack of conflict should not have occurred in a generalized way because in the Theta-EEG group there was also a Stroop effect, although it was minor may have occurred in some subjects and in some trials, which is supported by the greater variance observed in this group in the behavioral outcomes.

All of these findings indicate that people with an excess of theta activity show a peculiar ERP pattern that can be observed across multiple levels of the neurocognitive system before any clinical sign of cognitive impairment is observed, supporting the idea that aging is not a homogeneous process; therefore, there are probably several subgroups within the “healthy” elderly population. This study is the first to clearly exhibit a preclinical cognitive deficit in healthy elderly subjects with an excess of theta in their EEG.

## Author contributions

TF made substantial contributions to the conception of the work, EEG analysis, and interpretation of the data; she drafted parts of the manuscript and revised it critically; SS-M made substantial contributions to design of the work, ERPs acquisition and analysis, and interpretation of data; he drafts the first version of the manuscript and revised subsequent versions; GA-C made substantial contributions to the ERPs acquisition and analysis, and interpretation of data; she revised the manuscript critically; JS-P made substantial contributions to the statistical analysis and interpretation of data; he drafted parts of the manuscript and revised it critically; SG-S made substantial contributions to the interpretation of data; she revised the manuscript critically; JS-L made substantial contributions to design of the work and to interpretation of data; he made the figures and revised the manuscript critically; GO-O made substantial contributions to the interpretation of data and revised the manuscript critically; SS-M, GA-C, JS-P, SG-S, JS-L, GO-O, and TF give their final approval of the version to be published and agree to be responsible for all aspects of the work so that questions about the accuracy or integrity of any part of the work are adequately investigated and solved.

### Conflict of interest statement

The authors declare that the research was conducted in the absence of any commercial or financial relationships that could be construed as a potential conflict of interest. The reviewer GG and handling Editor declared their shared affiliation, and the handling Editor states that the process nevertheless met the standards of a fair and objective review.

## References

[B1] Alain C. Woods D. L. (1999). Age-related changes in processing auditory stimuli during visual attention: evidence for deficits in inhibitory control and sensory memory. Psychol. Aging 14, 507–519. 10.1037/0882-7974.14.3.507 10509703

[B2] Anderson E. D. Wahoske M. Huber M. Norton D. Li Z. Koscik R. L.. (2016). Cognitive variability—a marker for incident MCI and AD: an analysis for the Alzheimer's Disease Neuroimaging Initiative. Alzheimers Dement. 4, 47–55. 10.1016/j.dadm.2016.05.003 27489880 PMC4961828

[B3] Babiloni C. Binetti G. Cassetta E. Dal Forno G. Del Percio C. Ferreri F.. (2006). Sources of cortical rhythms change as a function of cognitive impairment in pathological aging: a multicenter study. Clin. Neurophysiol. 117, 252–268. 10.1016/j.clinph.2005.09.019 16377238

[B4] Babor T. F. Higgins-Biddle J. C. Saunders J. B. Monteiro M. G. (2001). Audit. The Alcohol Use Disorders Identification Test. Guidelines for Use in Primary Care. Geneva: World Health Association.

[B5] Baltes P. B. Lindenberger U. (1997). Emergence of a powerful connection between sensory and cognitive functions across the adult life span: a new window to the study of cognitive aging? Psychol. Aging 12, 12–21. 10.1037/0882-7974.12.1.12 9100264

[B6] Beck A. T. Ward C. H. Mendelson M. Mock J. Erbaugh J. (1961). An inventory for measuring depression. Arch. Gen. Psychiatry 4, 561–571. 10.1001/archpsyc.1961.01710120031004 13688369

[B7] Berry A. S. Shah V. D. Baker S. L. Vogel J. W. O'Neil J. P. Janabi M. (2016). Aging affects dopaminergic neural mechanisms of cognitive flexibility. J. Neurosci. 14, 12559–12569. 10.1523/JNEUROSCI.0626-16.2016 PMC5157103 27807030

[B8] Bielak A. A. M. Hultsch D. F. Strauss E. MacDonald S. W. S. Hunter M. A. (2010). Intraindividual variability is related to cognitive change in older adults: evidence for within-person coupling. Psychol. Aging 25, 575–586. 10.1037/a0019503 20853965

[B9] Binder J. C. Bezzola L. Haueter A. I. Klein C. Kühnis J. Baetschmann H.. (2017). Expertise-related functional brain network efficiency in healthy older adults. BMC Neurosci. 18:2. 10.1186/s12868-016-0324-1 28049445 PMC5209906

[B10] Buckner R. L. (2004). Memory and executive function in aging and AD: multiple factors that cause decline and reserve factors that compensate. Neuron 44, 195–208. 10.1016/j.neuron.2004.09.006 15450170

[B11] Bush G. Whalen P. J. Rosen B. R. Jenike M. A. McInerney S. C. Rauch S. L. (1998). The counting stroop: an interference task specialized for functional neuroimaging-validation study with functional MRI. Hum. Brain Mapp. 6, 270–282. 9704265 10.1002/(SICI)1097-0193(1998)6:4<270::AID-HBM6>3.0.CO;2-0 PMC6873370

[B12] Bush G. Whalen P. J. Shin L. M. Rauch S. L. (2006). The counting stroop: a cognitive interference task. Nat. Protoc. 1, 230–233. 10.1038/nprot.2006.35 17406237

[B13] Buzsáki G. (2006). Rhythms of the Brain. New York, NY: Oxford University Press.

[B14] Cabeza R. Anderson N. D. Locantore J. K. McIntosh A. R. (2002). Aging gracefully: compensatory brain activity in high-performing older adults. Neuroimage 17, 1394–1402. 10.1006/nimg.2002.1280 12414279

[B15] Castro-Chavira S. A. Barrios F. A. Pasaye E. H. Alatorre-Cruz G. C. Fernandez T. (2016). Compensatory larger cortical thickness in healthy elderly individuals with electroencephalographic risk for cognitive decline. Neuroreport 27, 710–715. 10.1097/WNR.0000000000000602 27171033

[B16] Chambers R. D. Griffiths S. K. (1991). Effects of age on the adult auditory middle latency response. Hear. Res. 51, 1–10. 10.1016/0378-5955(91)90002-Q 2013537

[B17] Chang B. S. Schomer D. L. Niedermeyer E. (2011). Normal EEG and sleep: adults and elderly, in Niedermeyer's Electroencephalography: Basic Principles, Clinical Applications, and Related Fields, eds Schomer D. L. Lopes da Silva F. H. (New York, NY: Wolters Kluwer, Lippincott Williams & Wilkins), 183–214.

[B18] Chen A. Bailey K. Tiernan B. N. West R. (2011). Neural correlates of stimulus and response interference in a 2-1 mapping Stroop task. Int. J. Psychophysiol. 80, 129–138. 10.1016/j.ijpsycho.2011.02.012 21356252

[B19] Coderre E. Conklin K. van Heuven W. J. B. (2011). Electrophysiological measures of conflict detection and resolution in the Stroop task. Brain Res. 1413, 51–59. 10.1016/j.brainres.2011.07.017 21840503

[B20] Davis S. W. Dennis N. A. Daselaar S. M. Fleck M. S. Cabeza R. (2008). Que PASA? the posterior-anterior shift in aging. Cereb. Cortex 18, 1201–1209. 10.1093/cercor/bhm155 17925295 PMC2760260

[B21] Dixon R. A. Garrett D. D. Lentz T. L. MacDonald S. W. S. Strauss E. Hultsch D. F. (2007). Neurocognitive markers of cognitive impairment: exploring the roles of speed and inconsistency. Neuropsychology 21, 381–399. 10.1037/0894-4105.21.3.381 17484601

[B22] Endicott J. Nee J. Harrison W. Blumenthal R. (1993). Quality of life enjoyment and satisfaction questionnaire: a new measure. Psychopharmacol. Bull. 29, 321–326. 8290681

[B23] Federmeier K. D. Kutas M. Schul R. (2010). Age-related and individual differences in the use of prediction during language comprehension. Brain Lang. 115, 149–161. 10.1016/j.bandl.2010.07.006 20728207 PMC2975864

[B24] Giaquinto S. Nolfe G. (1986). The EEG in the normal elderly: a contribution to the interpretation of aging and dementia. Electroencephalogr. Clin. Neurophysiol. 63, 540–546. 10.1016/0013-4694(86)90141-0 2422003

[B25] Gratton G. Coles M. G. H. Donchin E. (1983). A new method for off-line removal of ocular artifact. Electroencephalogr. Clin. Neurophysiol. 55, 468–484. 10.1016/0013-4694(83)90135-9 6187540

[B26] Grieve S. M. Williams L. M. Paul R. H. Clark C. R. Gordon E. (2007). Cognitive aging, executive function, and fractional anisotropy: a diffusion tensor MR imaging study. Am. J. Neuroradiol. 28, 226–235. 17296985 PMC7977408

[B27] Harada C. N. Natelson Love M. C. Triebel K. (2013). Normal cognitive aging. Clin. Geriatr. Med. 29, 737–752. 10.1016/j.cger.2013.07.002 24094294 PMC4015335

[B28] Hartikainen P. Soininen H. Partanen J. Helkala E. L. Riekkinen P. (1992). Aging and spectral analysis of EEG in normal subjects: a link to memory and CSF AChE. Acta Neurol. Scand. 86, 148–155. 10.1111/j.1600-0404.1992.tb05057.x 1414224

[B29] Hedden T. Gabrieli J. D. E. (2004). Insights into the ageing mind: a view from cognitive neuroscience. Nat. Rev. Neurosci. 5, 87–96. 10.1038/nrn1323 14735112

[B30] Hernández J. L. Valdés P. Biscay R. Virues T. Szava S. Bosch J.. (1994). A global scale factor in brain topography. Int. J. Neurosci. 76, 267–278. 10.3109/00207459408986009 7960483

[B31] Holtzer R. (2008). Within-person across-neuropsychological test variability and incident dementia. JAMA 300:823. 10.1001/jama.300.7.823 18714062 PMC2736784

[B32] Huang C. Wahlund L. Dierks T. Julin P. Winblad B. Jelic V. (2000). Discrimination of Alzheimer's disease and mild cognitive impairment by equivalent EEG sources:across-sectional and longitudinal study. Clin. Neurophysiol. 111, 1961–1967. 10.1016/S1388-2457(00)00454-5 11068230

[B33] Hultsch D. F. MacDonald S. W. Dixon R. A. (2002). Variability in reaction time performance of younger and older adults. J. Gerontol. B. Psychol. Sci. Soc. Sci. 57, 101–115. 10.1093/geronb/57.2.P101 11867658

[B34] Ila A. B. Polich J. (1999). P300 and response time from a manual Stroop task. Clin. Neurophysiol. 110, 367–373. 10.1016/S0168-5597(98)00053-7 10210626

[B35] Jelic V. Johansson S. E. Almkvist O. Shigeta M. Julin P. Nordberg A.. (2000). Quantitative electroencephalography in mild cognitive impairment: longitudinal changes and possible prediction of Alzheimer's disease. Neurobiol. Aging 21, 533–540. 10.1016/S0197-4580(00)00153-6 10924766

[B36] Kavcic V. Zalar B. Giordani B. (2016). The relationship between baseline EEG spectra power and memory performance in older African Americans endorsing cognitive concerns in a community setting. Int. J. Psychophysiol. 109, 116–123. 10.1016/j.ijpsycho.2016.09.001 27613569 PMC7202928

[B37] Larson M. J. Clayson P. E. Clawson A. (2014). Making sense of all the conflict: a theoretical review and critique of conflict-related ERPs. Int. J. Psychophysiol. 93, 283–297. 10.1016/j.ijpsycho.2014.06.007 24950132

[B38] Liotti M. Woldorff M. G. Perez R. Mayberg H. S. (2000). An ERP study of the temporal course of the stroop color-word interference effect. Neuropsychologia 38, 701–711. 10.1016/S0028-3932(99)00106-2 10689046

[B39] Lopes da Silva F. H. (2011). Neurocognitive processes and the EEG/MEG, in Niedermeyer's Electroencephalography: Basic Principles, Clinical Applications, and Related Fields, eds Schomer D. L. Lopes da Silva F. H. (New York, NY: Wolters Kluwer, Lippincott Williams & Wilkins), 1083–1112.

[B40] MacLeod C. M. (1991). Half a century of research on the stroop effect: an integrative review. Psychol. Bull. 109, 163–203. 10.1037/0033-2909.109.2.163 2034749

[B41] Mager R. Bullinger A. H. Brand S. Schmidlin M. Schärli H. Müller-Spahn F.. (2007). Age-related changes in cognitive conflict processing: an event-related potential study. Neurobiol. Aging 28, 1925–1935. 10.1016/j.neurobiolaging.2006.08.001 16973245

[B42] McDonald J. H. (2009). Handbook of Biological Statistics. Baltimore, MD: Sparky House Publishing.

[B43] Ostrosky-Solís F. Ardila A. Rosselli M. (1999). NEUROPSI: a brief neuropsychological test battery in Spanish with norms by age and educational level. J. Int. Neuropsychol. Soc. 5, 413–433. 10.1017/S1355617799555045 10439587

[B44] Park D. C. Smith A. D. Lautenschlager G. Earles J. L. Frieske D. Zwahr M.. (1996). Mediators of long-term memory performance across the life span. Psychol. Aging 11, 621–637. 10.1037/0882-7974.11.4.621 9000294

[B45] Patel S. H. Azzam P. N. (2005). Characterization of N200 and P300: selected studies of the event-related potential. Int. J. Med. Sci. 2, 147–154. 10.7150/ijms.2.147 16239953 PMC1252727

[B46] Prichep L. S. John E. R. Ferris S. H. Rausch L. Fang Z. Cancro R.. (2006). Prediction of longitudinal cognitive decline in normal elderly with subjective complaints using electrophysiological imaging. Neurobiol. Aging 27, 471–481. 10.1016/j.neurobiolaging.2005.07.021 16213630

[B47] Prichep L. S. John E. R. Ferris S. H. Reisberg B. Almas M. Alper K.. (1994). Quantitative EEG correlates of cognitive deterioration in the elderly. Neurobiol. Aging 15, 85–90. 10.1016/0197-4580(94)90147-3 8159266

[B48] Ramos-Goicoa M. Galdo-Alvarez S. Diaz F. Zurron M. (2016). Effect of normal aging and of mild cognitive impairment on event-related potentials to a stroop color-word task. J. Alzheimers. Dis. 52, 1487–1501. 10.3233/JAD-151031 27079705

[B49] Rayner K. Reichle E. D. Stroud M. J. Williams C. C. Pollatsek A. (2006). The effect of word frequency, word predictability, and font difficulty on the eye movements of young and older readers. Psychol. Aging 21, 448–465. 10.1037/0882-7974.21.3.448 16953709

[B50] Rebai M. Bernard C. Lannou J. (1997). The stroop's test evokes a negative brain potential, the N400. Int. J. Neurosci. 91, 85–94. 10.3109/00207459708986367 9394217

[B51] Reisberg B. Ferris S. H. De Leon M. J. Crook T. (1988). Global deterioration scale (GDS). Psychopharmacol. Bull. 24, 661–663. 3249768

[B52] Reisberg B. Ferris S. H. Kluger A. Franssen E. Wegiel J. de Leon M. J. (2008). Mild cognitive impairment (MCI): a historical perspective. Int. Psychogeriatr. 20, 18–31. 10.1017/S1041610207006394 18031593

[B53] Roca-Stappung M. Fernández T. Becerra J. Mendoza-Montoya O. Espino M. Harmony T. (2012). Healthy aging: relationship between quantitative electroencephalogram and cognition. Neurosci. Lett. 510, 115–120. 10.1016/j.neulet.2012.01.015 22266305

[B54] Román Lapuente F. Sánchez Navarro J. P. (1998). Cambios neuropsicológicos asociados al envejecimiento normal. An. Psicol. 14, 27–42.

[B55] Salthouse T. A. (1996). The processing-speed theory of adult age differences in cognition. Psychol. Rev. 103, 403–428. 10.1037/0033-295X.103.3.403 8759042

[B56] Salthouse T. A. (2000). Aging and measures of processing speed. Biol. Psychol. 54, 35–54. 10.1016/S0301-0511(00)00052-1 11035219

[B57] Samson R. D. Barnes C. A. (2013). Impact of aging brain circuits on cognition. Eur. J. Neurosci. 37, 1903–1915. 10.1111/ejn.12183 23773059 PMC3694726

[B58] Schneider Bakos D. Pinheiro de Paula Couto M. C. Vieira Melo W. Parente M. A. de M. P. Koller S. H. Bizarro L. (2008). Executive functions in the young elderly and oldest old: a preliminary comparison emphasizing decision making. Psychol. Neurosci. 1, 183–189. 10.3922/j.psns.2008.2.011

[B59] Soininen H. Partanen J. Paakkonen A. Koivisto E. Riekkinen J. (1991). Changes in absolute power values of EEG spectra in the follow-up of Alzheimer's disease. Acta Neurol. Scand. 83, 133–136. 10.1111/j.1600-0404.1991.tb04662.x 2017898

[B60] Spieler D. H. Balota D. A. Faust M. E. (1996). Stroop performance in healthy younger and older adults and in individuals with dementia of the Alzheimer's type. J. Exp. Psychol. Hum. Percept. Perform. 22, 461–479. 10.1037/0096-1523.22.2.461 8934854

[B61] Stroop J. R. (1935). Studies of interference in serial verbal reactions. J. Exp. Psychol. 18, 643–662. 10.1037/h0054651

[B62] Thomas A. K. Dave J. B. Bonura B. M. (2010). Theoretical perspectives on cognitive aging, in Handbook of Medical Neuropsychology, eds Armstrong C. L. Morrow L. (New York, NY: Springer), 297–313. Available online at: http://link.springer.com/chapter/10.1007/978-1-4419-1364-7_16

[B63] Tillman C. M. Wiens S. (2011). Behavioral and ERP indices of response conflict in Stroop and flanker tasks: response conflict in stroop and flanker tasks. Psychophysiology 48, 1405–1411. 10.1111/j.1469-8986.2011.01203.x 21457276

[B64] Valdés P. Biscay R. Galán L. Bosch J. Zsava S. Virués T. (1990). High resolution spectral EEG norms topography. Brain Topogr. 3, 281–282.

[B65] van der Hiele K. Bollen E. L. Vein A. A. Reijntjes R. H. Westendorp R. G. van Buchem M. A.. (2008). EEG markers of future cognitive performance in the elderly. J. Clin. Neurophysiol. 25, 83–89. 10.1097/WNP.0b013e31816a5b25 18340274

[B66] van der Hiele K. Vein A. A. Reijntjes R. H. A. M. Westendorp R. G. J. Bollen E. L. E. M. van Buchem M. A.. (2007). EEG correlates in the spectrum of cognitive decline. Clin. Neurophysiol. 118, 1931–1939. 10.1016/j.clinph.2007.05.070 17604688

[B67] van Veen V. Carter C. S. (2002). The timing of action-monitoring processes in the anterior cingulate cortex. J. Cogn. Neurosci. 14, 593–602. 10.1162/08989290260045837 12126500

[B68] Vaughan L. Giovanello K. (2010). Executive function in daily life: age-related influences of executive processes on instrumental activities of daily living. Psychol. Aging 25, 343–355. 10.1037/a0017729 20545419

[B69] Verhaeghen P. Steitz D. W. Sliwinski M. J. Cerella J. (2003). Aging and dual-task performance: a meta-analysis. Psychol. Aging 18, 443–460. 10.1037/0882-7974.18.3.443 14518807

[B70] Wechsler D. (2003). WAIS-III Escala Wechsler de Inteligencia Para adultos-III. 2nd Edn. México: Manual Moderno.

[B71] West R. (2003). Neural correlates of cognitive control and conflict detection in the Stroop and digit-location tasks. Neuropsychologia 41, 1122–1135. 10.1016/S0028-3932(02)00297-X 12667546

[B72] West R. (2004). The effects of aging on controlled attention and conflict processing in the Stroop task. J. Cogn. Neurosci. 16, 103–113. 10.1162/089892904322755593 15006040

[B73] West R. Alain C. (1999). Event-related neural activity associated with the Stroop task. Brain Res. Cogn. Brain Res. 8, 157–164. 10.1016/S0926-6410(99)00017-8 10407204

[B74] West R. Alain C. (2000). Age-related decline in inhibitory control contributes to the increased Stroop effect observed in older adults. Psychophysiology 37, 179–189. 10.1111/1469-8986.3720179 10731768

[B75] West R. Bailey K. Tiernan B. N. Boonsuk W. Gilbert S. (2012). The temporal dynamics of medial and lateral frontal neural activity related to proactive cognitive control. Neuropsychologia 50, 3450–3460. 10.1016/j.neuropsychologia.2012.10.011 23085124

[B76] West R. Jakubek K. Wymbs N. Perry M. Moore K. (2005). Neural correlates of conflict processing. Exp. Brain Res.. 167, 38–48. 10.1007/s00221-005-2366-y 16082533

[B77] Wlotko E. W. Federmeier K. D. Kutas M. (2012). To predict or not to predict: age-related differences in the use of sentential context. Psychol. Aging 27, 975–988. 10.1037/a0029206 22775363 PMC3685629

[B78] Yesavage J. A. Brink T. L. Rose T. L. Lum O. Huang V. Adey M.. (1983). Development and validation of a geriatric depression screening scale: a preliminary report. J. Psychiatr. Res. 17, 37–49. 10.1016/0022-3956(82)90033-4 7183759

[B79] Yuan P. Raz N. (2014). Prefrontal cortex and executive functions in healthy adults: a meta-analysis of structural neuroimaging studies. Neurosci. Biobehav. Rev. 42, 180–192. 10.1016/j.neubiorev.2014.02.005 24568942 PMC4011981

[B80] Zurrón M. Lindín M. Galdo-Alvarez S. Díaz F. (2014). Age-related effects on event-related brain potentials in a congruence/incongruence judgment color-word Stroop task. Front. Aging Neurosci. 6:128. 10.3389/fnagi.2014.00128 24987369 PMC4060640

[B81] Zurrón M. Pouso M. Lindín M. Galdo S. Díaz F. (2009). Event-related potentials with the Stroop colour-word task: timing of semantic conflict. Int. J. Psychophysiol. 72, 246–252. 10.1016/j.ijpsycho.2009.01.002 19171167

[B82] Zysset S. Schroeter M. L. Neumann J. Yves von Cramon D. (2007). Stroop interference, hemodynamic response and aging: an event-related fMRI study. Neurobiol. Aging 28, 937–946. 10.1016/j.neurobiolaging.2006.05.008 21887888

