# Supplementary material for: Two Different Populations within the Healthy Elderly: Lack of Conflict Detection in Those at Risk of Cognitive Decline
Source: Front Hum Neurosci. 2018 Jan 11;11:658. doi: 10.3389/fnhum.2017.00658 (PMC5768990; doi:10.3389/fnhum.2017.00658)
Supplement: Supplementary file 1 [file DataSheet1.doc]

**3.2. ERP results when electrodes were re-referenced to the average reference.**

**3.2.1. 150 - 300 ms**

No significant main effect of Group was observed for this time window (F(1, 42) = 1.66, p = 0.204, ƞ^2^p = 0.038), and the Group x Condition interaction was not significant (F (1, 42) = 1.90, p = 0.174, ƞ^2^p = 0.043) either. When the topography factor was included, no significant differences were found (Group x Condition x Coronal interaction: F < 1; Group x Condition x Sagittal interaction: F < 1). The Group x Condition x Coronal x Sagittal interaction was also not significant (F(16, 672) = 2.156, p = 0.074, ɛ = 0.257, ƞ^2^p = 0.049).

**3.2.2. 300 - 500 ms**

The analysis of the amplitude in this time window showed no differences between groups (F (1, 42) = 2.581, p = 0.116, ƞ^2^p = 0.058), or in the Group x Condition interaction (F < 1), in the Group x Condition x Coronal interaction (F < 1) or in the Group x Condition x Sagittal interaction (F < 1). Nevertheless, there was a significant Group x Condition x Coronal x Sagittal interaction (F(16, 672) = 3.054, p = 0.014, ɛ = 0.287, ƞ^2^p = 0.068). Post-hoc comparisons showed that for several electrodes, the effect was significant in both groups. In the Normal-EEG group, significant differences (p < 0.05) between the conditions were observed in F7, Fz, FT7, FC3, FCz, FT8, T3, C3, Cz, T4, CP3, CPz, TP8, and Pz, while in the Theta-EEG group, significant differences (p < 0.05) were observed in F3, Fz, FC3, FCz, T3,C3, Cz, TP7, CP3, CPz, and Pz (see Table S1).

| **Table S1.** P300 (300-500 ms) post-hoc comparisons of Group x Condition x Coronal x Sagittal interaction. | | | | |
| --- | --- | --- | --- | --- |
| Coronal | Sagittal | Electrode | Normal-EEG | Theta-EEG |
|  |  |  | (MD) | (MD) |
| Frontal | Left | F7 | 0.74* | 0.04 |
|  | Left-medial | F3 | 0.37 | 0.71** |
|  | Medial | Fz | 1.15*** | 0.61* |
|  | Right medial | F4 | 0.41 | 0.22 |
|  | Right | F8 | 0.19 | 0.66 |
| Fronto-central | Left | FT7 | 0.60** | 0.28 |
|  | Left-medial | FC3 | 0.54* | 0.61* |
|  | Medial | FCz | 1.09*** | 0.71* |
|  | Right-medial | FC4 | 0.40 | 0.30 |
|  | Right | FT8 | 0.60* | 0.11 |
| Central | Left | T3 | 0.53** | 0.40* |
|  | Left-medial | C3 | 0.50* | 0.60* |
|  | Medial | Cz | 0.88** | 0.58* |
|  | Right-medial | C4 | 0.45 | 0.17 |
|  | Right | T4 | 0.80* | 0.02 |
| Centro-parietal | Left | TP7 | 0.31 | 0.57** |
|  | Left-medial | CP3 | 0.50* | 0.52** |
|  | Medial | CPz | 0.50* | 0.58* |
|  | Right-medial | CP4 | 0.33 | 0.31 |
|  | Right | TP8 | 0.83* | 0.18 |
| Parietal | Left | T5 | 0.20 | 0.30 |
|  | Left-medial | P3 | 0.30 | 0.33 |
|  | Medial | Pz | 0.47* | 0.48* |
|  | Right-medial | P4 | 0.20 | 0.38 |
|  | Right | T6 | 0.55 | 0.03 |
| *p < 0.05, **p < 0.01, ***p < 0.001.  MD: Mean difference. | | | | |

**3.2.3. 500 - 700 ms**

In this time window, no significant main effect of Group (F(1, 42) = 3.100, p = 0.086, ƞ^2^p = 0.069) nor Group x Condition interaction (F < 1) were observed. The Group x Condition x Sagittal interaction was significant (F(4, 168) = 4.859, p = 0.010, ɛ = 0.492, ƞ^2^p = 0.104). Post-hoc comparisons showed that differences only were observed for the Normal-EEG group. The Normal-EEG group showed a significant interference effect in the Left (MD = 0.496 µV, p = 0.002), Left-medial (MD = -0.394 µV, p = 0.026), Medial (MD = -0.963 µV, p < 0.001), and Right-medial (MD = -0.477 µV, p < 0.001) sagittal regions. In contrast, the Theta-EEG group did not show a significant interference effect in any sagittal area. A similar pattern was observed for the significant Group x Condition x Coronal x Sagittal interaction (F(16, 672) = 3.277, p = 0.008, ɛ = 0.301, ƞ^2^p = 0.072).

Table S2 shows that the post-hoc comparisons between the stimuli for the N500 wave across electrodes over the scalp were significant for most of the locations in the Normal-EEG group, while for the Theta-EEG group, there was a significant difference only at the F4 electrode (p < 0.05).

| **Table S2.** N500 (500-700 ms) post-hoc comparisons of Group x Condition x Coronal x Sagittal interaction. | | | | |
| --- | --- | --- | --- | --- |
| Coronal | Sagittal | Electrode | Normal-EEG | Theta-EEG |
|  |  |  | (MD) | (MD) |
| Frontal | Left | F7 | -1.11*** | -0.25 |
|  | Left-medial | F3 | -0.33 | -0.04 |
|  | Medial | Fz | -0.26 | -0.12 |
|  | Right medial | F4 | -0.03 | -0.43* |
|  | Right | F8 | -0.60 | -0.72 |
| Fronto-central | Left | FT7 | -0.65** | -0.20 |
|  | Left-medial | FC3 | -0.26 | -0.11 |
|  | Medial | FCz | -1.00*** | -0.15 |
|  | Right-medial | FC4 | -0.30 | -0.06 |
|  | Right | FT8 | -0.56 | -0.50 |
| Central | Left | T3 | -0.51* | -0.16 |
|  | Left-medial | C3 | -0.75** | -0.29 |
|  | Medial | Cz | -1.44*** | -0.34 |
|  | Right-medial | C4 | -0.88*** | -0.13 |
|  | Right | T4 | -0.44 | -0.48 |
| Centro-parietal | Left | TP7 | -0.19 | -0.06 |
|  | Left-medial | CP3 | -0.80** | -0.27 |
|  | Medial | CPz | -1.15*** | -0.38 |
|  | Right-medial | CP4 | -0.77*** | -0.24 |
|  | Right | TP8 | -0.36 | -0.26 |
| Parietal | Left | T5 | -0.02 | -0.09 |
|  | Left-medial | P3 | -0.48* | -0.06 |
|  | Medial | Pz | -0.95*** | -0.13 |
|  | Right-medial | P4 | -0.42* | -0.11 |
|  | Right | T6 | -0.16 | -0.29 |
| *p < 0.05, **p < 0.01, ***p < 0.001.  MD: Mean difference. | | | | |
